# Neuropeptide Y and brain ageing: an updated scoping review on the role of autophagy

**DOI:** 10.3389/fnagi.2026.1888512

**Published:** 2026-09-21

**Authors:** Suang Yao Cheah, Yatinesh Kumari, Nur Fatin Nabilah Mohd Sahardi, Siti Liyana Saud Gany, Nur Atiqah Haizum Abdullah, Suzana Makpol, Faizul Jaafar

**Affiliations:** 1 Jeffrey Cheah School of Medicine and Health Sciences, Monash University Malaysia, Bandar Sunway, Selangor, Malaysia; 2 Secretariat of Research and Innovation, Faculty of Medicine, Universiti Kebangsaan Malaysia, Kuala Lumpur, Malaysia; 3 Department of Biochemistry, Faculty of Medicine, Universiti Kebangsaan Malaysia, Kuala Lumpur, Malaysia; 4 Department of Tissue Engineering and Regenerative Medicine, Faculty of Medicine, Universiti Kebangsaan Malaysia, Kuala Lumpur, Malaysia

**Keywords:** autophagy, brain aging, cognitive decline, neuropeptide Y, neuroprotection

## Abstract

Brain ageing is a complex physiological process characterised by progressive structural and functional changes that often result in cognitive decline and reduced quality of life in the elderly. These changes are driven by multiple mechanisms, including synaptic dysfunction, oxidative stress, neuroinflammation, and impaired cellular homeostasis. Neuropeptides have emerged as important regulators of these processes. Among them, neuropeptide Y (NPY) has gained attention for its potential role in mitigating age-related brain dysfunction. However, the extent to which NPY contributes to brain ageing remains unclear. This scoping review aims to map the available evidence on the role of NPY in brain ageing, focusing on the effect of NPY on autophagy, a key mechanism involved in brain ageing. Scientific studies published between 2014 and 2026 relevant to the research topic were systematically screened and reviewed. Following a rigorous selection process, four articles that met the inclusion and exclusion criteria were included for analysis. The findings indicate that NPY levels decline in the hypothalamic arcuate nucleus in a progeroid mouse model, where restoring NPY delays several ageing phenotypes. In addition, NPY appears to mitigate brain ageing by promoting autophagy in the hypothalamus and cerebral cortex, an effect requiring both suppression of PI3K/AKT/mTOR signalling and activation of ERK1/2-MAPK. These insights may pave the way for future research into therapeutic strategies targeting NPY to combat age-related cognitive deficits and neurodegenerative diseases. However, further investigation is needed to elucidate the underlying mechanisms of NPY action, including the roles of its various receptors and interactions with other neuropeptides.

## Graphical abstract

**Figure:**
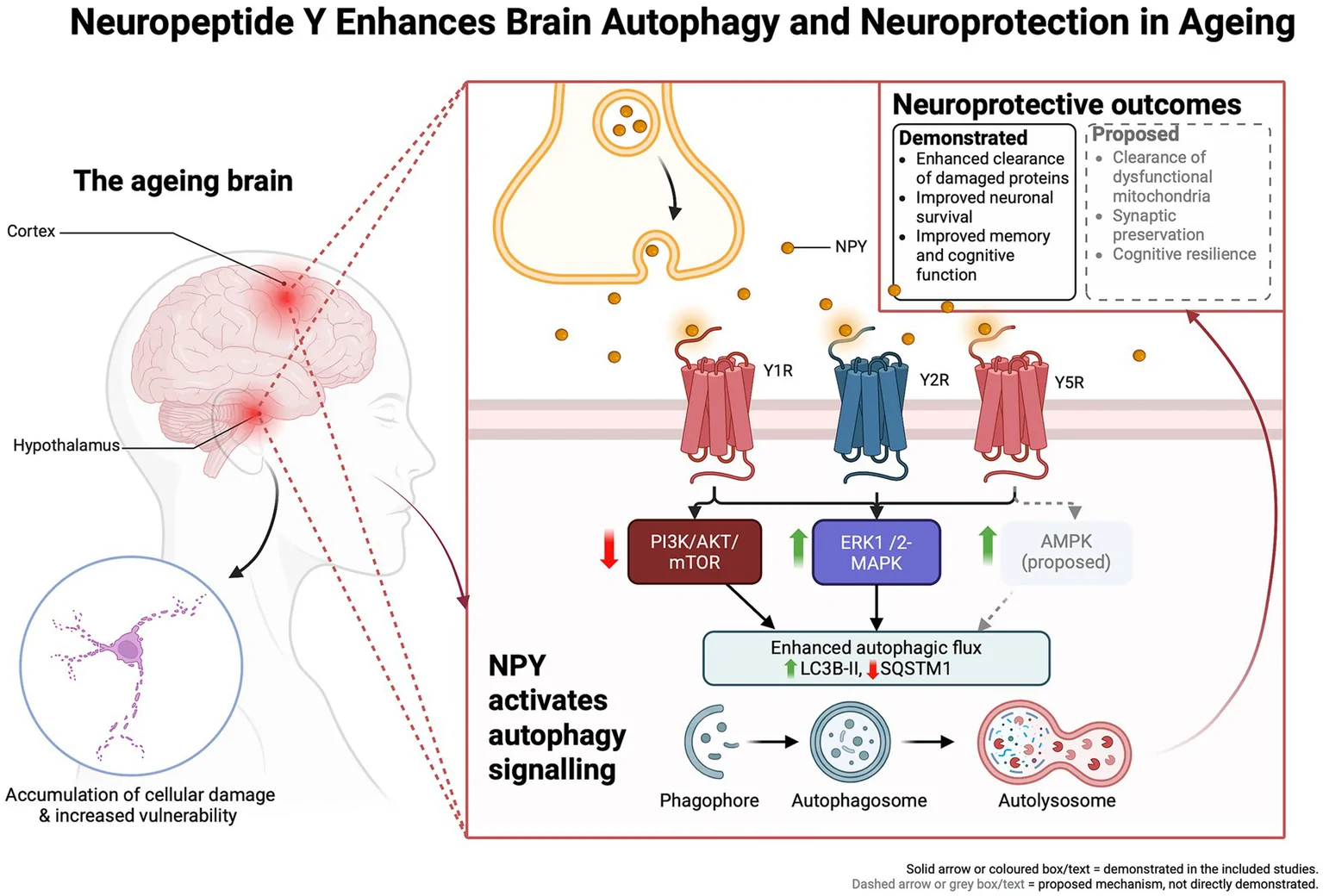

## 1 Introduction

Ageing is a complex and irreversible biological process characterised by the gradual decline of physiological function and the reduced ability to maintain homeostasis. This process is driven by 12 hallmarks, namely genomic instability, telomere attrition, epigenetic alterations, loss of proteostasis, disabled macroautophagy, deregulated nutrient sensing, mitochondrial dysfunction, cellular senescence, stem cell exhaustion, altered intercellular communication, chronic inflammation and dysbiosis (López-Otín et al., 2023). Ageing affects all organ systems, but its impact on the brain is particularly profound (Smith et al., 2024). As part of the nervous system, the brain plays a central role in cognition, behaviour, and systemic regulation. It is highly vulnerable to age-related changes due to its high metabolic demand (Rae et al., 2024), limited regenerative capacity (Groh and Simons, 2025), and sensitivity to oxidative (Șerban et al., 2025) and metabolic stress (Elzinga et al., 2025). Among the hallmarks, impaired macroautophagy is particularly relevant to brain ageing because neurons are long-lived, post-mitotic cells that depend on continuous cellular quality-control mechanisms to remove damaged proteins and organelles. The progressive loss of autophagic efficiency may therefore contribute substantially to declining neuronal resilience and function during ageing.

Autophagy comprises three principal forms: macroautophagy, microautophagy, and chaperone-mediated autophagy (CMA) (Wang et al., 2023). Macroautophagy is the most extensively studied and is commonly referred to simply as autophagy throughout this text (Yao and Shen, 2023). The brain is composed primarily of neurons and glial cells, both of which possess distinct autophagic pathways that contribute to overall neural function (Jimenez-Sanchez et al., 2022). Autophagy plays a critical role in clearing misfolded proteins, defective mitochondria, and synaptic components in neurons. These processes are essential for sustaining synaptic plasticity and neurotransmission (Jimenez-Sanchez et al., 2022). However, neuronal autophagy becomes progressively less efficient during ageing, leading to the accumulation of toxic protein aggregates such as β-amyloid and phosphorylated tau (Stavoe and Holzbaur, 2019). Glial cells (astrocytes, microglia, and oligodendrocytes) also rely on autophagy to regulate energy metabolism, neuroinflammation, and myelin maintenance. With advancing age, glial autophagy declines, resulting in exaggerated inflammatory responses, impaired phagocytic activity, and reduced trophic support for neurons (Wang et al., 2024).

The autophagy machinery is activated by various cellular stressors such as nutrient deprivation, oxidative stress, hypoxia, or the accumulation of misfolded proteins (Palmer et al., 2025). The process is primarily regulated by two nutrient-sensing kinases: mammalian target of rapamycin (mTOR) and AMP-activated protein kinase (AMPK). These kinases exert opposing effects on the Unc-51-like kinase 1 (ULK1) complex, which represents the initiating step of autophagy. While AMPK activates ULK1 complex under energy-deprived conditions, mTOR suppresses its activity in nutrient-rich states. The ULK1 complex consists of FAK family kinase-interacting protein of 200 kDa (FIP200), autophagy-related protein 13 (ATG13), and autophagy-related protein 101 (ATG101) (Figure 1). Upon activation, ULK1 stimulates the class III phosphatidylinositol 3-kinase (PI3KC3) complex to initiate phagophore nucleation, followed by a cascade of ATG protein, microtubule-associated protein 1 light chain 3 (LC3) family proteins and complexes activation responsible for membrane elongation, autophagosome maturation, fusion with lysosomes, and ultimately degradation of the cargo (Palmer et al., 2025). Interestingly, recent studies have revealed that autophagy is also modulated by neuroendocrine signals (Catalani et al., 2017). Among these, neuropeptide Y (NPY) has emerged as a critical regulator linking energy homeostasis and neuronal survival to autophagic activity (Feng et al., 2024).

**Figure 1:**
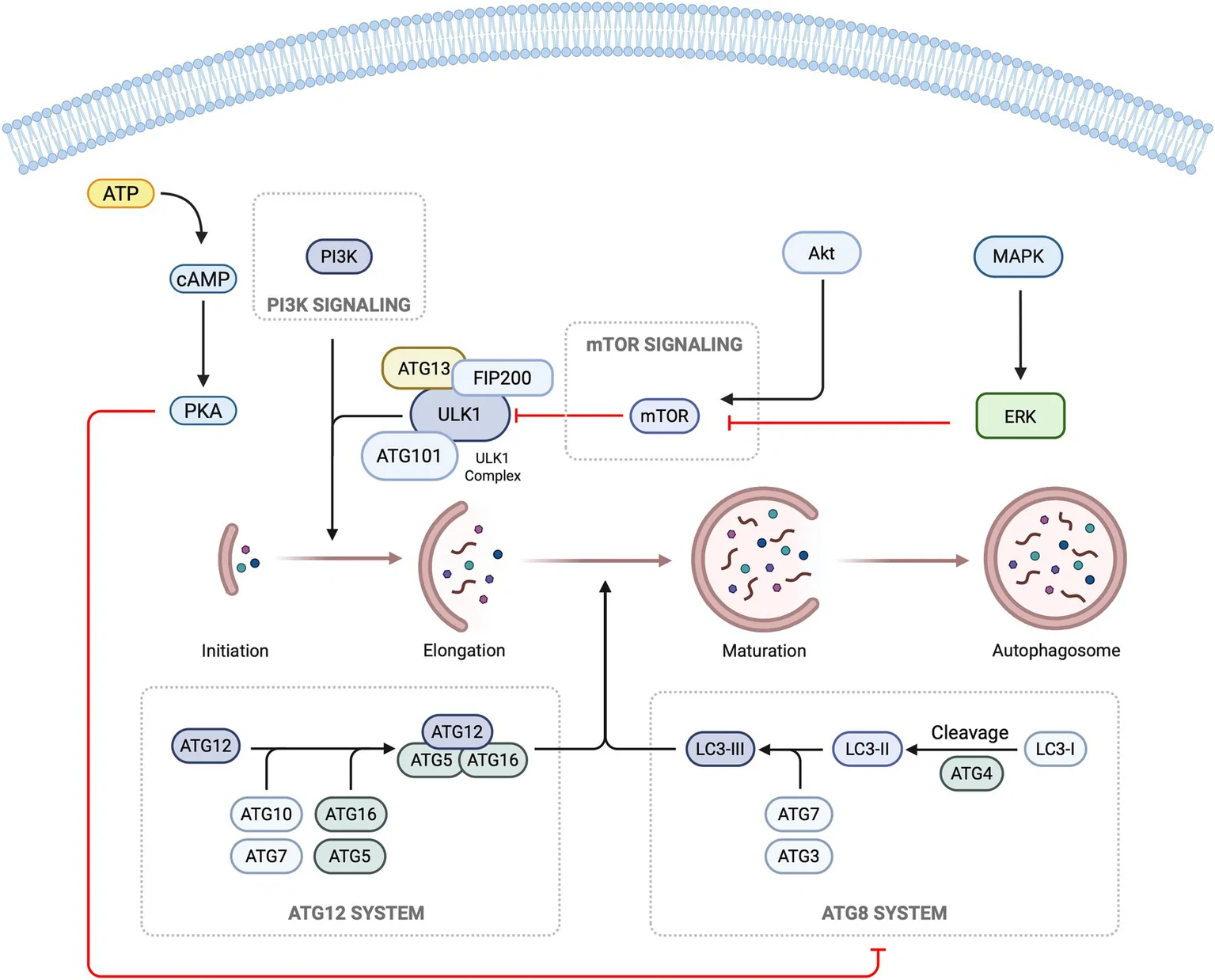
Schematic representation of autophagy signalling pathways regulation. Figure created by the authors based on the concepts described in Palmer et al. (2025) with BioRender.com.

NPY is a highly conserved 36-amino-acid peptide and among the most abundant neuropeptides in the mammalian brain (Park et al., 2022). NPY is widely expressed in the hypothalamus, hippocampus, cortex, and limbic system particularly gamma-aminobutyric acid (GABA)-ergic interneurons. Its effects are mediated by G-protein-coupled Y receptors: Y1, Y2, Y4 and Y5 are functional in humans, whereas Y6 is a truncated, non-functional gene product in humans and other primates (though functional in rodents); Y1, Y2 and Y5 regulate feeding behaviour, while Y1, Y2 and Y4 modulate anxiety and depression (Park et al., 2022; Tang et al., 2022). Additionally, studies have shown that NPY is able to cross the blood–brain barrier (BBB) (Nelson et al., 2024). In the peripheral circulation, NPY also acts as a multifunctional regulator influencing energy balance, glucose metabolism, and lipid storage (Lee and Herzog, 2021). These biological processes are mediated through its interaction with Y receptors in peripheral tissues such as adipose tissue, liver, and the cardiovascular system. Despite all these biological processes, NPY has also been shown to play a crucial role in neural health where it demonstrates neuroprotective properties (Stéphanie et al., 2022). It has been shown to reduce excitotoxicity, inhibit apoptosis, modulate neuroinflammation, and enhance neurogenesis. NPY is among the neuropeptides for which direct receptor- and pathway-specific evidence of autophagic regulation has been reported in hypothalamic and cortical neurons. This combination of age-associated changes, broad physiological functions and experimentally demonstrated effects on autophagic flux provides a strong rationale for examining NPY in the context of brain ageing. Nevertheless, the present review does not assume that NPY is more important than all other neuropeptides.

Through activation of receptors such as Y1 and Y5, NPY influences the mTOR and AMPK pathways, thereby promoting clearance of misfolded proteins and damaged organelles (Lanjewar et al., 2025). This NPY–autophagy axis may represent a vital defence mechanism against protein aggregation, oxidative stress, and mitochondrial dysfunction that drive brain ageing and neurodegeneration. Despite these advances, the relationship between NPY, autophagy, and brain ageing remains incompletely understood. While several narrative reviews have addressed NPY’s neuroprotective roles, few have systematically synthesised evidence linking it to autophagy regulation in the context of brain ageing. The available literature is fragmented, with studies focusing on different receptor subtypes, brain regions, and disease models. A scoping review is therefore needed to integrate these findings, map the mechanistic insights, and identify research gaps. By bringing together evidence from *in vitro*, *in vivo*, and translational studies, this review aims to clarify the NPY–autophagy–ageing axis and assess its therapeutic potential for mitigating neurodegenerative disorders associated with ageing.

## 2 Methodology

A scoping review methodology was selected, rather than a systematic review, because the available literature on NPY and autophagy in brain ageing is limited and methodologically heterogeneous. The objective of this review is therefore to map the breadth and nature of existing evidence and to identify knowledge gaps (evidence mapping), rather than to pool or statistically synthesise comparable outcomes across studies (evidence synthesis). This scoping review was conducted based on the framework developed by Arksey and O’Malley (2005) and it was conducted based on Preferred Reporting Items for Systematic Reviews and Meta-analyses Extension for Scoping Reviews (Tricco et al., 2018). The process involved the following steps: 1. formulating research question, 2. collecting relevant studies, 3. screening and selecting studies, 4. extracting relevant data, and 5. presenting the findings.

### 2.1 Define research question

A Population, Concept, and Context (PCC) framework was employed to formulate the review question. The Population comprised ageing biological systems, including older human adults, aged animal models, models of genetically accelerated (progeroid) ageing, and senescent neuronal and glial cell models, with younger age groups included where they served as comparators. The Concept was the role of neuropeptide Y (NPY) in brain ageing physiology, encompassing NPY expression, tissue and circulating levels, receptor subtypes (Y1, Y2, and Y5), and downstream signalling, with particular emphasis on autophagy and proteostasis, as well as related outcomes including cognition, memory, neuroinflammation, adult neurogenesis, and neuronal survival. The Context comprised *in vivo*, *ex vivo*, *in vitro*, and human clinical or post-mortem studies of the ageing central nervous system in the absence of a defined neurodegenerative disease, without geographical restriction.

Accordingly, the overarching question guiding this review was: In the ageing brain (Population), how does neuropeptide Y (Concept) modulate brain ageing physiology through autophagy across experimental and human studies (Context)? This was operationalised into two specific research questions: 1. Through which NPY receptors and downstream signalling pathways does NPY regulate autophagy in the neurons? and 2. What functional and neuroprotective outcomes are associated with NPY-mediated autophagy modulation in brain ageing?

### 2.2 Search strategies and data sources

A comprehensive literature search was conducted to identify relevant studies published from January 2014 to July 2026 using three electronic databases: PubMed, Scopus, and Web of Science. The search string was developed around three key concepts: Neuropeptide Y (NPY), brain ageing, and autophagy. The general search string was:

(“Neuropeptide Y” OR NPY) AND (aging OR ageing OR “brain aging” OR “brain ageing” OR senescence OR “healthy aging” OR “aging brain” OR “neuronal aging” OR “cognitive aging”) AND (autophagy OR autophagic OR autophagosome OR lysosome OR lysosomal OR mitophagy OR macroautophagy OR microautophagy OR “chaperone-mediated autophagy”).

Boolean operators AND and OR were used to combine search terms, and database-specific syntax and indexing terms were applied as appropriate. The full search string for each database is provided in Supplementary Table S1. The final search was executed on 31 July 2026. No review protocol was registered prospectively. This review was reported in accordance with the PRISMA-ScR checklist (Tricco et al., 2018), which is provided in full as Supplementary Table S2.

### 2.3 Study selection

The selection of articles was conducted in two stages. Firstly, two researchers (CSY and FJ) independently screened the titles and abstracts based on the inclusion and exclusion criteria. Irrelevant abstracts were removed, and full-text articles retrieved for those that met the selection criteria. In the second stage, the full-text articles were independently reviewed by both researchers to determine their relevance to the objectives of the review. Any disagreements regarding article selection were resolved through discussion with a third party (NAHA) until a consensus was reached. The search results were managed using the Covidence software.

#### 2.3.1 Inclusion criteria

Studies were eligible for inclusion if they met all of the following criteria: (1) published between January 2014 and July 2026; (2) conducted in human participants or rodent models; (3) examined brain ageing in the absence of a defined neurodegenerative disease, including comparisons of aged with young or adult groups, models of genetically accelerated (progeroid) ageing, and ageing-relevant interventions such as caloric restriction; (4) investigated neuropeptide Y, its receptors, or its downstream signalling as a primary or explicitly reported variable; (5) reported original empirical findings, whether *in vivo*, ex vivo, or *in vitro*; (6) available in full text; and (7) published in English.

#### 2.3.2 Exclusion criteria

Studies were excluded if they met any of the following criteria: (1) published outside the period January 2014 to July 2026; (2) conducted in species other than humans or rodents; (3) investigated a defined neurodegenerative disease or acute neurological insult, including Alzheimer’s disease, Parkinson’s disease, Huntington’s disease and other neurodegenerative conditions, or models of stroke, epilepsy, or traumatic brain injury; (4) did not report data on neuropeptide Y, its receptors, or its signalling; (5) were secondary or non-empirical publications, including narrative reviews, systematic reviews, meta-analyses, editorials, commentaries, policy papers, and book chapters; (6) were conference abstracts, preprints, theses, or otherwise unavailable in full text; and (7) not published in English.

Neurodegenerative disease models were excluded to preserve the distinction between brain ageing itself and disease-specific neurodegeneration. Although the two processes share mechanisms such as oxidative stress, impaired proteostasis and neuroinflammation, established neurodegenerative disorders contain additional disease-specific pathological features and interventions that may independently alter NPY signalling and autophagic activity. Disease-related terminology was therefore not used to exclude records during database retrieval. Instead, eligibility was determined during full-text screening according to the biological model and study objective. Genetically accelerated (progeroid) ageing models were retained, as they recapitulate features of normal brain ageing without superimposing a defined neurodegenerative pathology; where such a model is the source of evidence, this is stated explicitly.

### 2.4 Data extraction

A data extraction and synthesis protocol was developed based on the study objectives. The following information was extracted from each selected study: 1. Study characteristics including the first author, year of publication, species of study subjects, and study design, 2. specific areas of investigation related to NPY and brain ageing, and 3. key findings and interpretations relevant to the research focus. Data charting was performed by one reviewer (CSY) and verified by a second reviewer (FJ). Consistent with scoping review methodology, no formal critical appraisal of methodological quality was undertaken. This structured approach allowed comparison of individual studies and a detailed analysis of NPY’s role in brain ageing.

## 3 Results

### 3.1 Search results

A total of 69 studies were retrieved from PubMed, Scopus, and Web of Science. After removing 30 duplicates, 25 studies were excluded based on the exclusion criteria. Then, 10 studies were further excluded following full-text review. In the end, four studies fulfilled the inclusion criteria. The process of study selection is outlined in Figure 2.

**Figure 2:**
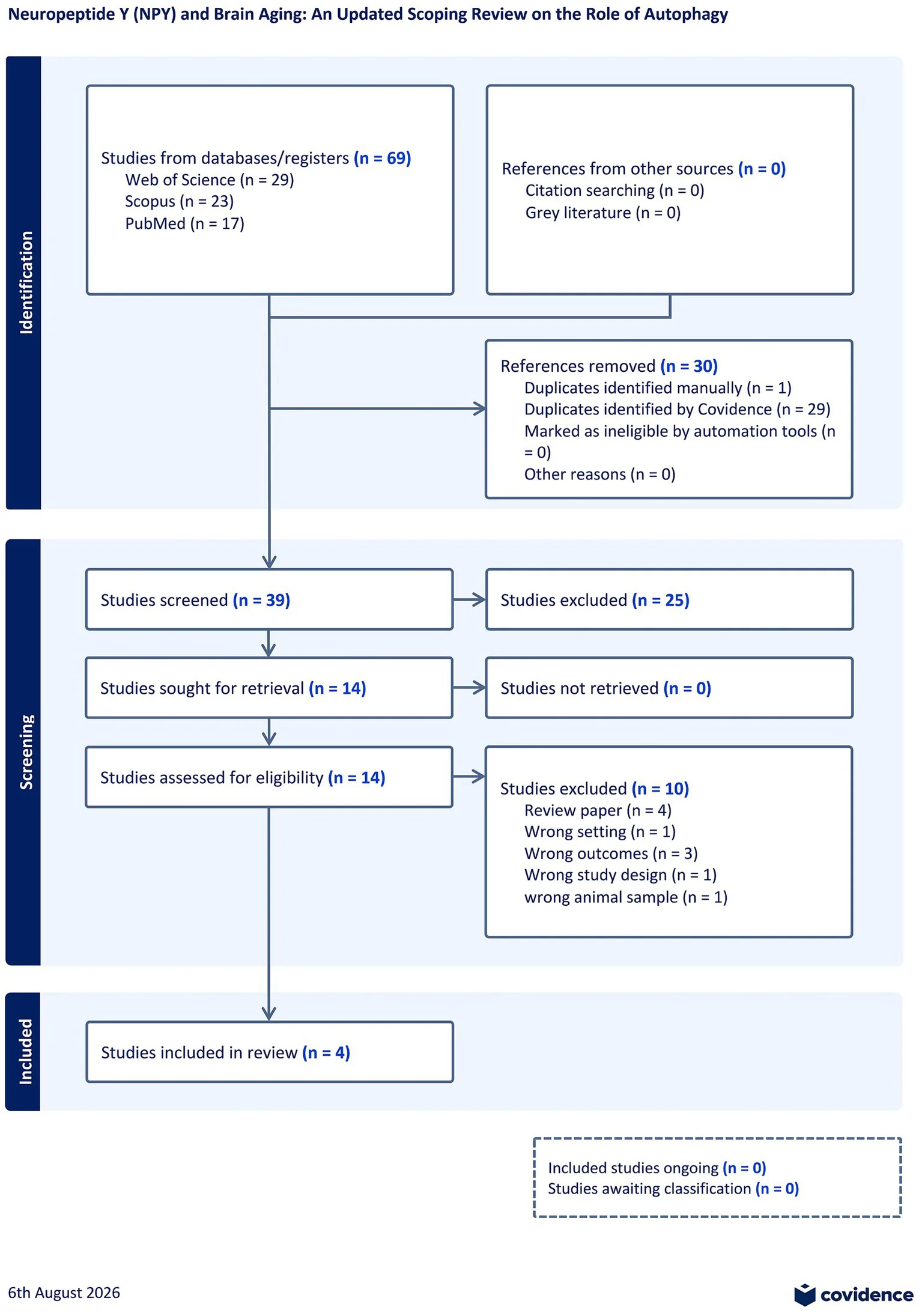
PRISMA flow diagram describing the search strategy and study selection process.

### 3.2 Study characteristics

Based on the area of focus, the four selected articles investigated the effects of NPY on autophagy in the hypothalamus and cerebral cortex during brain ageing (Aveleira et al., 2015; Ferreira-Marques et al., 2016; Ferreira-Marques et al., 2025; Ferreira-Marques et al., 2021). All four studies used rodent models and employed a combination of *in vivo* and *in vitro* study designs.

### 3.3 Effects of NPY on brain ageing

This scoping review recognised NPY as a pleiotropic neuropeptide with a significant role in the brain ageing process. The proposed neuroprotective effect of NPY in mitigating brain ageing is primarily attributed to its ability to stimulate autophagy. NPY has a stimulatory effect on autophagy in hypothalamic neurons as demonstrated by both *in vivo* and *in vitro* studies (Aveleira et al., 2015). The *in vitro* study used mHypoN42 cells, an embryonic mouse hypothalamic cell line, alongside primary differentiated hypothalamic neural cells derived from Wistar rat embryos. Treatment of both cell types with recombinant NPY (100 nM) increased the expression of LC3B-II, a key autophagy marker indicative of autophagosome formation. In addition, NPY treatment also increased autophagic flux in mHypoN42 cells, as evidenced by an increased number of autophagosomes and autolysosomes detected in tandem mCherry-GFP-LC3 cell-based assay.

This finding is further supported by SQSTM1 turnover under lysosomal blockade: the reduction in SQSTM1 normally seen with NPY treatment did not occur when chloroquine (an autolysosomal degradation inhibitor) was added to the cell culture medium, indicating that NPY lowers SQSTM1 via increased lysosomal degradation rather than reduced synthesis (Aveleira et al., 2015). The stimulatory effect of NPY on autophagy was reported to be achieved through NPY Y1 and Y5 receptors activation. Additionally, phosphatidylinositol 3-kinase (PI3K), extracellular signal-regulated kinase (ERK) and protein kinase A (PKA) signalling pathways were found to be closely related in the process of NPY-stimulated autophagy. Similar findings were observed in an *in vivo* experiment using transgenic male C57BL/6 mice overexpressing NPY in the hypothalamic arcuate nucleus (ARC) via adeno-associated viral vector-mediated gene transfer. Compared to the control group, these mice exhibited a decline in LC3B-II and SQSTM1 protein levels. This result suggests accelerated degradation of the marker proteins in the mouse hypothalamus, indicating a higher autophagic flux (Aveleira et al., 2015). Given the role of NPY in mediating autophagy, the study proposed NPY as a potential therapeutic option in treating ageing-associated decline in brain function (Aveleira et al., 2015).

Apart from hypothalamus, NPY has also been shown to mediate autophagy in cortical neurons, as demonstrated in an *in vitro* study (Ferreira-Marques et al., 2016). In this study, primary embryonic cortical neurons isolated from pregnant female Wistar rats were cultured and treated with NPY (100 nM). As a result, autophagic activity and autophagosome formation in the cells were higher than the control group, as indicated by elevated LC3B puncta immunoreactivity and LC3B-II protein levels. Furthermore, a reduction in SQSTM1 level (indicating an increased autolysosomal degradation) in the rat cortical neurons further supported this finding. NPY Y1, Y2 or Y5 receptors were demonstrated to play a role in NPY-induced autophagy in rat cortical neurons. Additionally, NPY was found to stimulate autophagy by inhibiting mechanistic target of rapamycin (mTOR), a kinase that suppresses autophagy (Ferreira-Marques et al., 2016). Interestingly, the study also investigated the role of ghrelin in autophagy regulation and found that it enhances autophagy in cortical neuron by upregulating NPY levels and activating NPY receptors. Based on the reviewed articles, the findings of studies investigating the effects of NPY treatment on brain ageing are comprehensively summarised and presented in Table 1.

**Table 1:** Characteristics and key findings of the included studies examining NPY, its receptors, and autophagy regulation in neuronal and rodent models relevant to brain ageing.

Authors (Year) [ref]	Study type (*in vivo* / *in vitro* studies)	Ageing model type	Brain region/origin of cell	Treatment/intervention	Key findings
Aveleira et al. (2015)	*in vitro*	Embryonic-derived neuronal culture; no ageing comparison	Hypothalamic neurons	Primary Treatments: Exposure to CR conditions Treatment with: Recombinant NPY NPY Y1 receptor agonists NPY Y5 receptor agonists Additional Drugs Used: Chloroquine NPY receptor antagonists (Y1, Y2, and Y5) PI3K inhibitors.	CR → autophagy in hypothalamic neurons. NPY → autophagy by activating Y1 and Y5 receptors. NPY-induced autophagy in hypothalamic neurons is mediated through activation of PI3K, ERK and PKA. NPY receptor antagonist could ⊣ the autophagy stimulated by CR in Hypothalamic Neurons
	*in vivo*	Adult mice, AAV-mediated NPY overexpression; no ageing comparison	Hypothalamus	Gene Transfer via AAV Vectors: Adeno-associated viral vectors carrying: *NPY* gene (to stimulate *NPY* overexpression) Green fluorescent protein gene (as a control). Driven by a neuronal-specific promoter Injected into the hypothalamic arcuate nucleus of mice	NPY induced autophagy in mouse hypothalamus
Ferreira-Marques et al. (2016)	*in vitro*	Embryonic primary neurons; no ageing comparison	Cortical neurons	Intervention: Nutrient deprivation to mimic caloric restriction Treatment with: NPY Acylated ghrelin Other drugs and receptor antagonists added: Chloroquine (lysosomal protein degradation inhibitor) NPY receptor antagonists (Y1, Y2, Y5) Ghrelin receptor antagonists	CR stimulated autophagy and ↑ of NPY mRNA, ghrelin mRNA and protein levels in the cells. CR-induced autophagy was mediated by NPY Y1, Y2 and Y5 receptor activation. NPY induced autophagy in primary rat cortical neurons through a similar pathway as CR. Ghrelin ↑ NPY mRNA and NPY protein levels Ghrelin enhanced autophagy in cortical neurons by ↑ NPY levels and stimulating NPY receptors activation. NPY Y1, Y2 and Y5 receptor antagonists ⊣ the stimulation of CR on autophagy
Ferreira-Marques et al. (2021)	*in vitro*	Embryonic primary neurons; no ageing comparison	Cerebral cortex (cortical neurons)	Primary Treatments: Exposure to CR conditions (low-glucose DMEM, 6 h) Treatment with: NPY (100 nM) Acylated ghrelin (10 nM) Additional Drugs Used: PI3K/AKT inhibitor (LY294002) ERK1/2-MAPK inhibitor (U0126)	CR, NPY and ghrelin → autophagy in cortical neurons (↑ LC3B-II, ↓ SQSTM1). CR, NPY and ghrelin ↓ phospho-AKT and ↓ phospho-mTOR, indicating autophagy stimulation through PI3K/AKT/mTOR inhibition. PI3K/AKT inhibitor ⊣ the NPY-induced ↑ LC3B-II and ↓ SQSTM1, confirming that PI3K/AKT/mTOR inhibition is required for NPY-induced autophagy. CR, NPY and ghrelin ↑ phospho-ERK; ERK1/2-MAPK inhibitor ⊣ this effect and partially ⊣ NPY-induced autophagy, indicating that ERK1/2-MAPK activation is also required. Crosstalk between pathways: PI3K/AKT inhibition ⊣ ERK phosphorylation under all stimuli. Under CR, AKT acts upstream of ERK; under NPY and ghrelin the two cascades are interdependent, as ERK1/2-MAPK inhibition partially reversed the ↓ in phospho-AKT. Under ERK1/2-MAPK inhibition, ghrelin-induced SQSTM1 degradation was impaired (↑ SQSTM1). Findings support NPY and ghrelin as CR mimetics acting on autophagy in cortical neurons.
Ferreira-Marques et al. (2025)	*in vivo*	Genetically accelerated (progeroid) ageing; not naturally aged	Hypothalamus (arcuate nucleus, ARC)	Gene Transfer via AAV Vectors: AAV1/2 vector carrying the *NPY* gene under a neuron-specific human synapsin promoter (3.6 × 10^9^ v.g. per side) Bilateral stereotaxic injection into the hypothalamic ARC; saline as vehicle control Pair-feeding, to control for the orexigenic effect of NPY Additional Assessments: Y-maze and open field tests at 30, 60, 90 and 120 days post-injection Endpoint at 4 months post-injection (immunohistochemistry, Western blotting, histology of skin, liver, heart and kidney)	Z24-KO mice showed ↓ NPY, ↓ NeuN, ↑ GFAP and ↓ Iba1 immunoreactivity in the ARC compared with age-matched wild-type mice, recapitulating ageing-related hypothalamic changes. AAV-mediated NPY overexpression produced a sustained (4-month) ↑ in NPY that was restricted to the ARC. Restoration of hypothalamic NPY → ↑ NeuN, ↓ GFAP, ↓ phospho-IκBα (⊣ NF-κB activation) and ↓ Tau, indicating neuroprotection and reduced neuroinflammation. ↓ SQSTM1 and ↓ LC3B-II in the hypothalamus, consistent with ↑ autophagic flux. ↑ body weight to wild-type levels from 30 to 90 days post-injection despite pair-feeding, and reversal of lipodystrophy. ↑ spatial recognition memory in the Y-maze, with no change in locomotor activity in the open field test. ↓ fur loss (alopecia) scores at all time points. Peripheral benefits: thicker epidermis and hypodermis with ↑ collagen, PCNA and KRT1 in skin; improved liver structure with ↑ PCNA, ↓ phospho-p53 and ↓ immune cell infiltration; ↓ cardiomyocyte hypertrophy; no change in kidney structure.

CR, caloric restriction, NPY, Neuropeptide Y, PI3K, Phosphatidylinositol 3-kinase; ERK, Extracellular signal-regulated kinase, PKA, Protein kinase A, ↑, increase; ↓, decrease; ⊣, inhibit; →, promote.

The intracellular machinery through which NPY activity is transduced has also been characterised in a neuronal model relevant to ageing. In primary cortical neurons obtained from embryonic day 18 to 19 Wistar rat embryos, caloric restriction, NPY (100 nM) and acylated ghrelin (10 nM) each increased LC3B-II and decreased SQSTM1, and all three stimuli reduced the phosphorylation of AKT and mTOR while increasing the phosphorylation of ERK1/2 (Ferreira-Marques et al., 2021). Pharmacological inhibition of PI3K with LY294002 abolished the NPY-induced rise in LC3B-II and the accompanying fall in SQSTM1, whereas inhibition of ERK1/2-MAPK with U0126 produced only partial blockade, indicating that NPY-stimulated autophagy requires both suppression of the PI3K/AKT/mTOR cascade and activation of ERK1/2-MAPK (Ferreira-Marques et al., 2021). The hierarchy of the two cascades differed according to the stimulus. Under caloric restriction, AKT appeared to act upstream of ERK, because PI3K inhibition prevented the caloric restriction-induced increase in ERK phosphorylation whereas ERK1/2 inhibition did not alter AKT phosphorylation; in contrast, under NPY or ghrelin stimulation the two pathways appeared to operate at the same level while remaining interdependent (Ferreira-Marques et al., 2021). A divergence between the two caloric restriction mimetics was also apparent, in that the ghrelin-induced decrease in SQSTM1 was independent of PI3K and was reversed by ERK1/2 inhibition, under which SQSTM1 accumulated (Ferreira-Marques et al., 2021). It should be noted that this study used embryonic rather than aged neurons and did not quantify NPY across age; its contribution is therefore mechanistic, defining how NPY activity is transduced rather than how that activity changes as the brain ages.

Evidence for an age-associated decline in hypothalamic NPY has been extended to a mammalian model of accelerated ageing. In *Zmpste24*^−^/^−^ (Z24-KO) progeroid mice, NPY immunoreactivity quantified across the anterior–posterior extent of the hypothalamic arcuate nucleus (ARC) was significantly lower than in age-matched wild-type mice (Ferreira-Marques et al., 2025). This reduction occurred alongside a fall in the neuronal marker NeuN, an increase in the gliosis marker GFAP and a decrease in the microglial marker Iba1, indicating that the loss of NPY in the ARC forms part of a broader profile of neuronal depletion, astrogliosis and altered innate immune status (Ferreira-Marques et al., 2025). Restoring hypothalamic NPY by bilateral stereotaxic delivery of an AAV1/2-hSyn-NPY vector into the ARC produced an approximately twofold, ARC-restricted increase in NPY that was sustained for 4 months and reversed several of these changes: NeuN was restored, while GFAP, phosphorylated IκBα and Tau were reduced, consistent with attenuated NF-κB activation and Tau pathology (Ferreira-Marques et al., 2025). Hypothalamic SQSTM1 and LC3B-II both decreased, which the authors interpreted as accelerated autophagic turnover rather than reduced autophagosome formation (Ferreira-Marques et al., 2025). Functionally, treated animals showed improved novel-arm exploration in the Y-maze with no change in open-field locomotor activity, indicating a specific gain in spatial recognition memory rather than a non-specific increase in activity. Because *Zmpste24* deficiency models accelerated rather than natural chronological ageing, and because the wild-type comparators were male and younger than the progeroid groups, these observations require confirmation in naturally aged animals (Ferreira-Marques et al., 2025).

## 4 Discussion

This scoping review analysed four studies, encompassing both *in vivo* rodent models and *in vitro* experimental systems. The findings highlight the emerging role of NPY in regulating autophagy involved in brain ageing. Across the reviewed studies, NPY consistently emerged as a significant modulator of ageing-related mechanisms including neuronal autophagy, neurogenesis, and synaptic plasticity across different brain regions (Aveleira et al., 2015; Ferreira-Marques et al., 2016; Ferreira-Marques et al., 2025; Ferreira-Marques et al., 2021). In the following sections, this review discusses in detail the effect of brain ageing on NPY expression and the potential impact of NPY treatment on ageing-related neuronal changes. Particular emphasis is placed on the role of autophagy in mediating these processes. Autophagy may represent a central mechanism linking NPY signalling with neuronal maintenance, cellular survival, and functional integrity during ageing. Collectively, these observations suggest that NPY may represent a promising therapeutic target for mitigating brain ageing through the modulation of autophagic pathways.

### 4.1 The effect of NPY on brain ageing and the role of autophagy

#### 4.1.1 NPY as a key regulator of autophagy

NPY acts as a neuroprotective agent, and its presence or therapeutic restoration may counteract several major drivers of brain ageing. This NPY peptide has been found to stimulate autophagy in neurons of the hypothalamus and brain cortex. These findings suggest a potential role in mitigating brain ageing from an autophagic perspective (Aveleira et al., 2015; Ferreira-Marques et al., 2016). Age-related downregulation of autophagy markers may contribute to autophagic dysfunction, resulting in the accumulation of harmful misfolded proteins and damaged organelles during ageing (Caponio et al., 2022). In addition, calorie restriction (CR) is an effective strategy for promoting cellular longevity and slowing the progression of structural and functional deterioration in brain ageing. This effect is primarily mediated through its stimulatory influence on autophagy (López-Otín et al., 2023; Mattson and Arumugam, 2018; Zhang et al., 2021). Consistent with this, studies in this review also found that activation of NPY and its receptors plays a key role in triggering autophagy in the brain in response to calorie restriction (Aveleira et al., 2015; Ferreira-Marques et al., 2016). Key signalling pathways interacting with NPY receptors to regulate this mechanism include mTOR, PI3K, ERK and PKA (Figure 3) (Aveleira et al., 2015; Ferreira-Marques et al., 2016). This aligns with previous literature highlighting the potential pro- or anti-ageing properties and neuroprotective roles of these proteins. Their effects are primarily mediated through involvement in cellular hallmarks of ageing, regulation of synaptic plasticity, reduction of neuroinflammation, and promotion of cellular longevity (Enns and Ladiges, 2010; Liu et al., 2023; Papadopoli et al., 2019; Sun and Nan, 2017).

**Figure 3:**
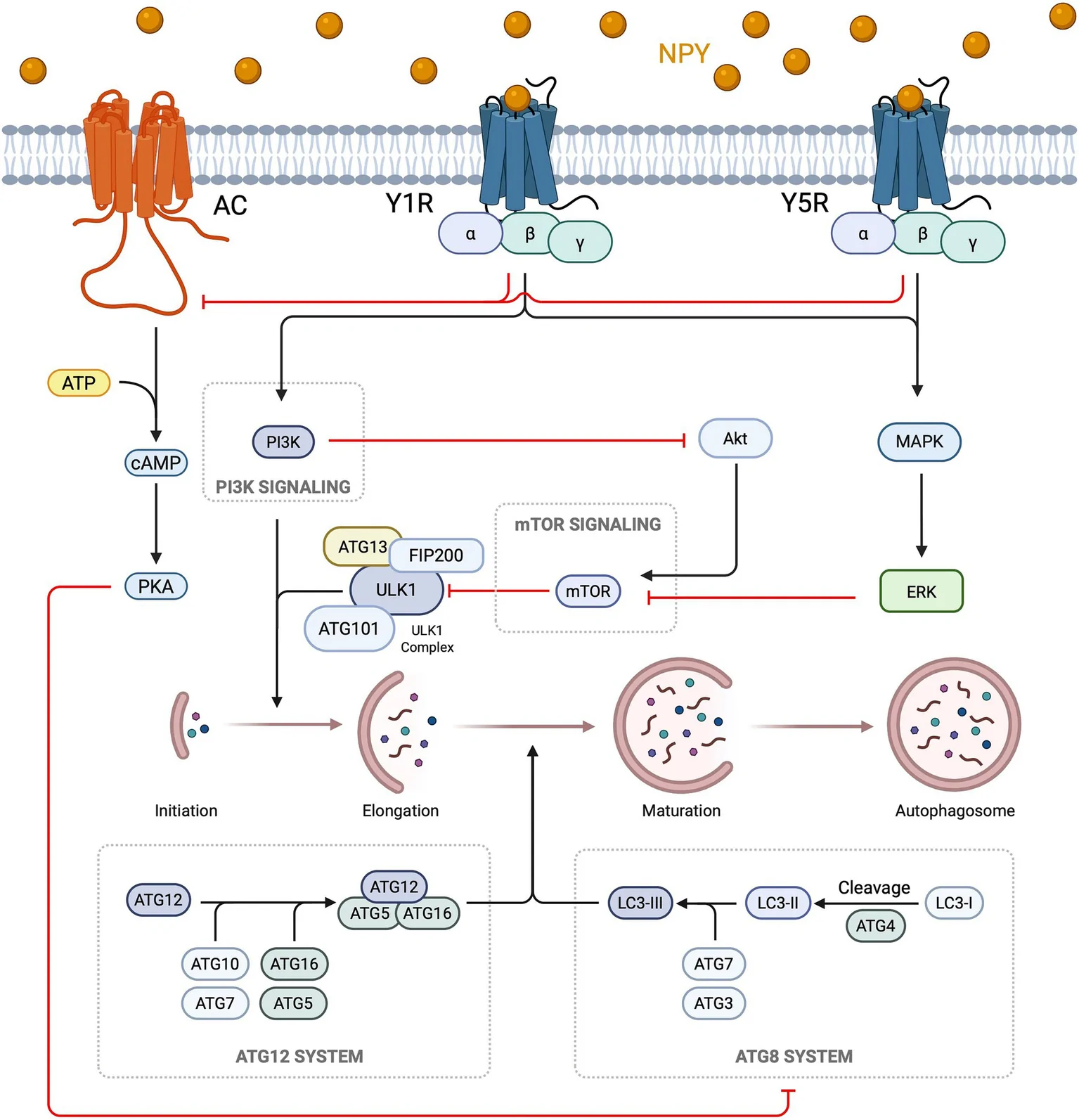
Mechanism of NPY-mediated activation of autophagy pathways. Created with BioRender.com.

#### 4.1.2 The role of NPY and NPY receptor in autophagy sex-dependent differences

Several articles have previously reported on sex differences in the effect of NPY in different contexts, such as anxiety attenuation, oestrogen-mediated NPY signalling, energy balance and insulin resistance on NPY-expressing neurons (Eva et al., 2020; Nahvi and Sabban, 2020). It has been reported that female rodents carry fewer NPY neurons and thus have lower NPY concentrations than males (Figure 4) (Nahvi and Sabban, 2020). Clinically, recent studies suggest that cognitive decline progresses more steeply in females than males, though socioeconomic factors may also contribute to these differences (Jin et al., 2023; Levine et al., 2021; Wolfova et al., 2024). They suggest that women may experience faster cognitive decline even though they initially have greater cognitive abilities (Levine et al., 2021). Hence, understanding sex-based variations in NPY activity is crucial for future research on its role in brain ageing and potential therapeutic application.

**Figure 4:**
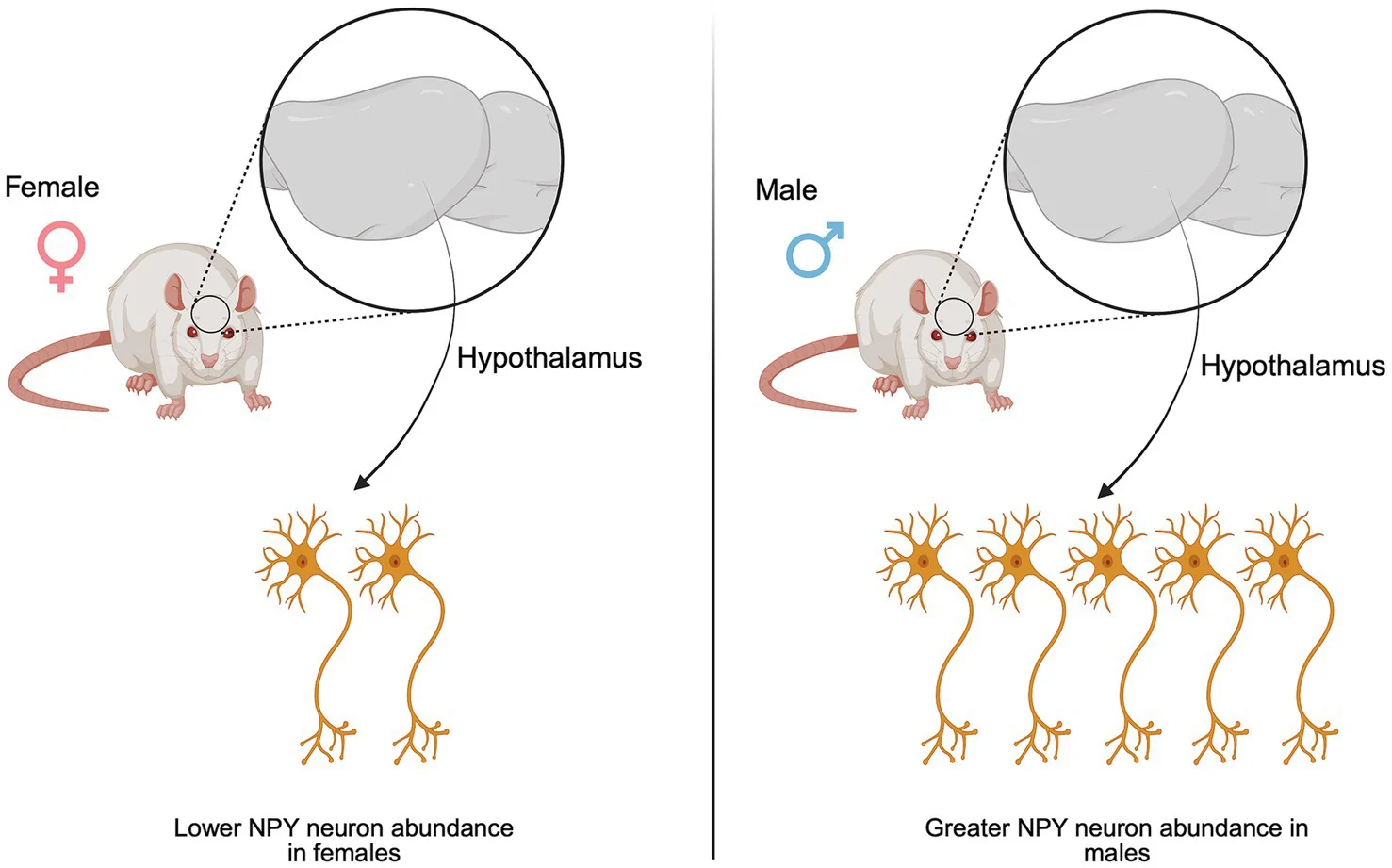
Sex differences in NPY neuron density. Created with BioRender.com.

Autophagy has been shown to be regulated by metabolic and hormonal signals that may differ between males and females (Wei and Huang, 2019; Shang et al., 2021). Oestrogen, for instance, has been shown to modulate autophagy through the activation of AMP-activated protein kinase and inhibition of mammalian target of rapamycin signalling pathways in neuronal cells (Wei and Huang, 2019; Zhao et al., 2024). These pathways overlap with those activated by NPY receptor signalling, suggesting potential convergence between sex hormones and NPY-mediated metabolic regulation (Aveleira et al., 2015). Previous studies further indicate that oestrogen influences the expression of NPY and NPY receptors and the responsiveness of hypothalamic neurons (Acosta-Martinez et al., 2007) and cancer cells (Amlal et al., 2006) to metabolic cues. In contrast, the role of androgen signalling in the regulation of NPY remains unclear and has not yet been fully determined. Consequently, differences in oestrogen and androgen signalling may alter how NPY regulates intracellular pathways associated with nutrient sensing and cellular stress responses. Such variations may affect autophagic activity in brain regions involved in memory and stress regulation, including the hippocampus and hypothalamus. Differences in neuronal autophagy efficiency have been proposed as one contributing factor to the distinct trajectories of brain ageing observed between males and females (Congdon, 2018). These observations suggest that NPY-mediated activation of autophagy may operate differently between sexes due to interactions with sex hormone signalling pathways.

#### 4.1.3 The role of NPY on autophagic regulation of the hypothalamic–pituitary–adrenal (HPA) axis

NPY appears to act as a functional counterbalance to CRH: it is co-localised in stress-responsive brain regions (hypothalamus, amygdala and hippocampus) and counteracts CRH-induced anxiety. Intracerebroventricular or local injection of NPY reduces anxiety in rodents, whereas NPY knockout mice display enhanced anxiety (Reichmann and Holzer, 2016). The concept that NPY participates in termination of the stress response posits that NPY inhibits CRH neurons and dampens HPA axis activation (Reichmann and Holzer, 2016). Indeed, stress alters NPY expression in a region- and paradigm-dependent manner; acute restraint or foot shock stress can increase NPY mRNA in the arcuate nucleus and dentate gyrus, whereas chronic stress may decrease expression (Reichmann and Holzer, 2016). Notably, intranasal NPY administration before or after severe stress (single prolonged stress) prevents stress-induced HPA dysregulation and behavioural abnormalities, highlighting its potential as a therapeutic modulator of stress resilience.

Within the HPA axis, the glucocorticoids have been shown to induce endoplasmic reticulum (ER) stress in hypothalamic neurons (An et al., 2023). Sustained ER stress disrupts neuronal homeostasis and impairs insulin signalling pathways, thereby contributing to neuronal dysfunction and metabolic dysregulation (An et al., 2023). One mechanism linking metabolic regulation with neuronal stress responses involves autophagy. Previous studies demonstrate that activation of autophagy in hypothalamic neurons promotes lipid mobilisation and generates free fatty acids, which subsequently increase the expression of the orexigenic neuropeptide agouti-related protein (AgRP) and stimulate food intake (An et al., 2023). In contrast, inhibition of autophagy suppresses the fasting-induced increase in AgRP expression and reduces feeding behaviour. Autophagy also plays a crucial role in regulating other hypothalamic neuropeptides. Loss of autophagy in proopiomelanocortin (POMC) neurons reduces the production of α-melanocyte-stimulating hormone (α-MSH) (Kaushik et al., 2012) and promotes hypothalamic inflammation through activation of the pro-inflammatory IκB kinase β signalling pathway (Meng and Cai, 2011). Importantly, ageing has been shown to reduce hypothalamic autophagic activity, which disrupts POMC secretion and contributes to the development of age-associated metabolic syndrome (Kaushik et al., 2012). Given the established role of NPY in modulating both stress responses and hypothalamic metabolic pathways, it is plausible that NPY may regulate the activity of the HPA axis through modulation of autophagy-related processes.

#### 4.1.4 The role of NPY and autophagy in energy metabolism

Insulin receptors are co-expressed in NPY expressing neurons (Loh et al., 2017). These findings indicate that disruption of insulin signalling within NPY neuronal circuits alone is sufficient to impair hippocampal-dependent memory. Binding of NPY to Y1 or Y2 receptors activates intracellular signalling cascades involving inhibition of adenylyl cyclase and reduction of cyclic AMP levels (Sheriff et al., 1998). This signalling modulates neuronal excitability and regulates neurotransmitter release. In the hippocampus, NPY inhibits excessive glutamate release from presynaptic terminals, thereby preventing excitotoxic damage (Wang, 2005). This neuroprotective action is particularly important in ageing where metabolic stress increases neuronal vulnerability to excitotoxicity. Furthermore, NPY signalling influences metabolic pathways by interacting with AMP-activated protein kinase and mTOR pathways that sense cellular nutrient availability (Dong et al., 2025).

Autophagy represents a critical downstream mechanism that integrates metabolic signals with cellular quality control. During nutrient deprivation or metabolic stress, AMP-activated protein kinase inhibits mTOR and directly activates ULK1, thereby initiating autophagosome formation (Wong et al., 2013). Previous studies have demonstrated that NPY activates AMP-activated protein kinase while suppressing mTOR signalling (Catalani et al., 2017). Activation of AMP-activated protein kinase promotes phosphorylation of ULK1 and stimulates the formation of the class III phosphatidylinositol 3 kinase complex that initiates autophagosome nucleation and subsequently autophagy processes. Previous study has shown that glucose deprivation induces the accumulation of DEP domain containing mTOR interacting protein (DEPTOR) (Zhao et al., 2011). DEPTOR binds to and inhibits both mTORC1 and mTORC2 complexes, thereby enhancing autophagy. Therefore, this review postulates that NPY may contribute to DEPTOR accumulation, thereby enhancing the clearance of damaged cellular components, restoring cellular energy balance, and reducing oxidative stress. Disruption of this metabolic regulatory system contributes to cognitive decline, whereas preservation of NPY-mediated metabolic signalling and autophagic activity may provide neuroprotective benefits in the ageing brain.

### 4.2 Integrated NPY–autophagy–ageing mechanistic framework

The available evidence supports an integrated NPY–autophagy–ageing framework in which age-associated cellular disturbances and NPY-mediated protective signalling are interconnected rather than operating as independent processes (Figure 5). During brain ageing, increased oxidative stress, mitochondrial dysfunction, metabolic stress, neuroinflammation, and declining proteostasis may progressively impair neuronal homeostasis, synaptic plasticity, and cognitive function. Within this context, NPY may function as an endogenous neuroprotective signal through the activation of Y receptors expressed in the hypothalamus, cerebral cortex, and other ageing-sensitive brain regions.

**Figure 5:**
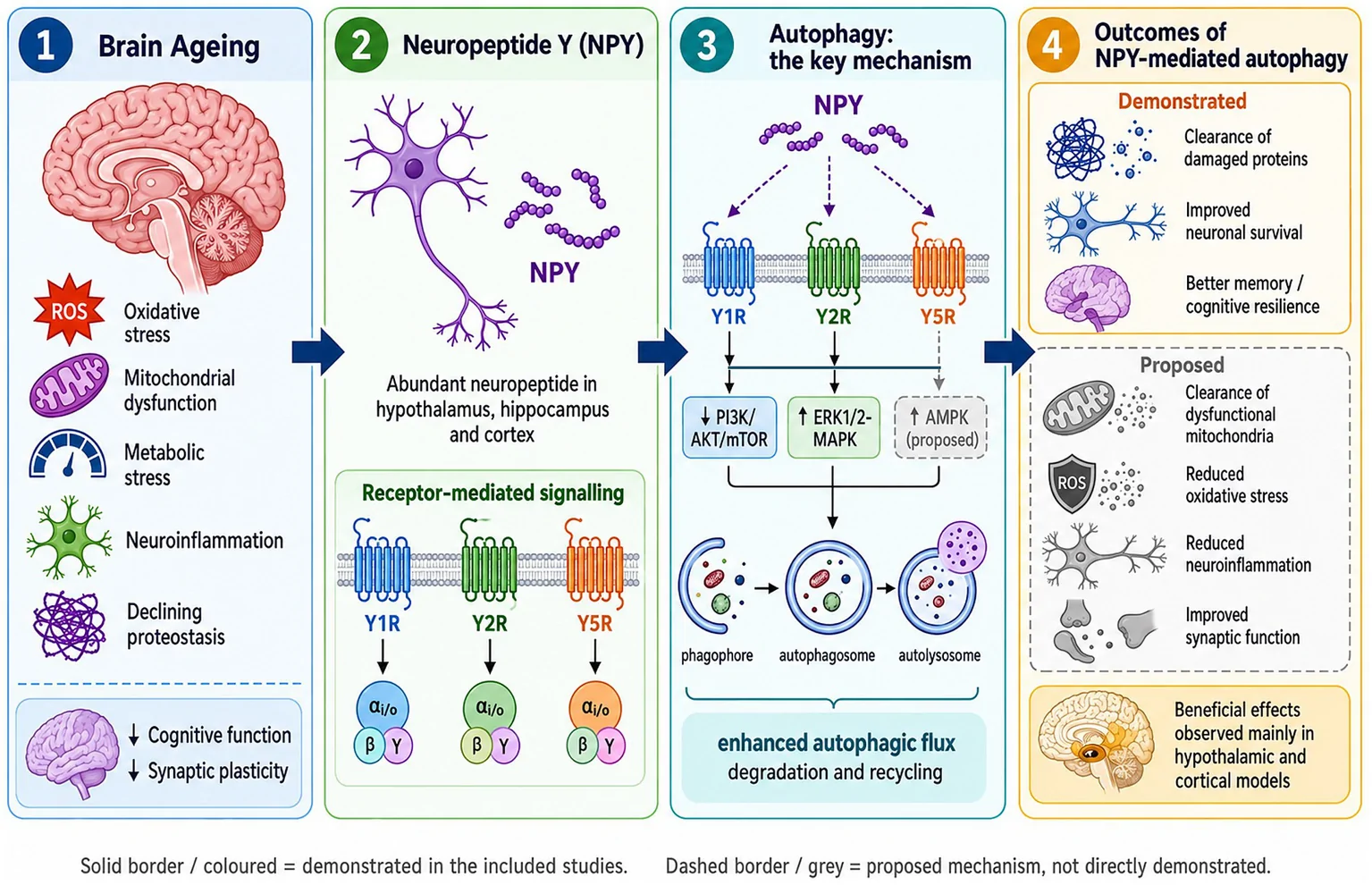
Integrated conceptual framework of the NPY–autophagy–ageing axis in the brain. Created with ChatGPT, OpenAI, version 5.6 Sol.

The included studies indicate that NPY-induced autophagy is mediated predominantly through Y1 and Y5 receptors in hypothalamic neurons and through Y1, Y2, and Y5 receptors in cortical neurons. Receptor activation is associated with suppression of the PI3K/AKT/mTOR pathway and activation of ERK1/2-MAPK signalling, thereby facilitating autophagosome formation, autolysosomal degradation, and enhanced autophagic flux. AMPK may provide an additional nutrient-sensing link between NPY signalling and autophagy, although its direct involvement in NPY-induced autophagy requires further experimental confirmation. Enhanced autophagic flux may promote the removal of damaged proteins and dysfunctional mitochondria, thereby limiting intracellular accumulation of toxic cellular components and reducing secondary oxidative and inflammatory responses. These effects may preserve neuronal viability, maintain synaptic integrity, and support cognitive resilience during ageing.

Consistent with this framework, restoration of hypothalamic NPY in a progeroid mouse model was associated with reduced SQSTM1 and LC3B-II levels, decreased neuroinflammatory signalling and Tau accumulation, preservation of neuronal markers, and improved spatial recognition memory. However, the proposed relationships between NPY-mediated autophagy and outcomes such as oxidative stress reduction, mitochondrial quality control, synaptic preservation, and cognitive resilience should be interpreted cautiously. Several components of this framework are derived from mechanistic integration across experimental studies rather than being demonstrated together within a single ageing model. Thus, Figure 5 presents a hypothesis-generating conceptual model that distinguishes the directly demonstrated NPY–receptor–autophagy pathway from its proposed downstream consequences, and highlights the need for validation in naturally aged animals, sex-balanced experimental designs, and human studies.

### 4.3 Translational considerations: potential dosing strategies and treatment regimens of NPY

Therapeutic strategies for NPY share one goal: to sustain NPY levels long enough to engage autophagy in the brain. The delivery approaches discussed below, including evidence from Alzheimer’s and Huntington’s disease models, fall outside this review’s inclusion criteria and are presented here solely as contextual, translational evidence rather than as findings of this scoping review. Lentiviral vectors encoding NPY fused to an apoB binding domain enable continuous systemic expression from the liver and spleen. In APP transgenic mice, a single injection reverses neurodegeneration, improves memory and increases neural precursor proliferation, without adverse effects over 3 months (Spencer et al., 2016). More directly relevant to autophagy, AAV mediated NPY delivery into the arcuate nucleus of prematurely ageing Z24-KO mice restores NPY for at least 4 months. This reduces NF-κB signalling and tau pathology, and notably increases autophagy markers, alongside improved memory and physical health (Ferreira-Marques et al., 2025). It also ameliorates skin and liver ageing. This suggests region-specific NPY supplementation acts partly by restoring autophagic capacity in the hypothalamus, linking NPY directly to the clearance mechanisms implicated in ageing. Intranasal NPY, tested in post-traumatic stress models, prevents stress-induced HPA dysregulation whether given before or after stress exposure. Limited human pilot data suggest a favourable safety profile, though whether this route sufficiently engages central autophagic pathways remains unclear (Reichmann and Holzer, 2016). Intracerebroventricular NPY and its fragments also show efficacy in Huntington’s disease and Alzheimer’s disease models. Their invasiveness reinforces the need for delivery methods, such as viral vectors or optimised intranasal formulations, that can sustain NPY exposure long enough to produce durable autophagic and neuroprotective effects.

Achieving these autophagy-related benefits is constrained by the pharmacokinetic profile of NPY. As a 36-amino-acid peptide, NPY has a very short systemic half-life. It is rapidly degraded by serum and endothelial peptidases, and further limited by blood–brain barrier proteolytic enzymes, so endogenous NPY persists for only a few minutes in circulation (Reichmann and Holzer, 2016). This poor stability explains why vector-based approaches, which drive continuous local expression, work best. They produce the sustained NPY exposure needed to upregulate autophagy. Peripheral peptide administration, by contrast, would require impractically frequent dosing unless modified through glycosylation or carrier conjugation (Reichmann and Holzer, 2016). Pharmacodynamically, NPY signals through Gi/o-coupled Y receptor subtypes. Y2 and Y5 activation inhibits presynaptic neurotransmitter release and reduces excitotoxicity, effects consistent with the AMPK and mTOR mediated autophagy induction described elsewhere in this review. Y1 activation, meanwhile, supports neurogenesis (Shapovalova et al., 2024). Understanding receptor distribution and downstream pathways will enable rational design of NPY mimetics that maximise neuroprotection while minimising undesired actions such as increased feeding or vasoconstriction. Receptor selective analogues could theoretically preserve these autophagy-promoting, neuroprotective actions while minimising side effects such as increased feeding. Future dosing regimens should therefore be designed around sustaining the NPY exposure needed to maintain autophagic flux, rather than replicating peptide levels alone.

### 4.4 Limitations

Several limitations should be highlighted in interpreting the results of this scoping review. All the included studies in this review were conducted using animal or cell models, *in vivo* or *in vitro*. Hence, the generalisability of these findings to human brain ageing is uncertain. Moreover, the studies varied in their experimental approaches and brain regions of focus, limiting the ability to compare the findings on the role of NPY directly across different studies and to form a definitive conclusion about the universal role of NPY across different neuronal populations. Because the included studies examined different brain regions (hypothalamus versus cortex) using different experimental paradigms, including caloric restriction, viral-mediated overexpression, and receptor antagonism, the apparent consistency of NPY’s autophagy-promoting effect may partly reflect convergent findings across distinct neuronal populations rather than a single unified mechanism; cross-study comparisons in this review should therefore be interpreted with this caveat in mind. Another limitation is that the number of studies included was small. It should also be noted that three of the four included studies originated from the same research group, so the findings await independent replication. In addition, the search was restricted to English-language publications and to three databases, data charting was performed by a single reviewer, and consistent with scoping review methodology, no formal critical appraisal of study quality was undertaken. No eligible human studies were identified, which is itself a notable gap. In addition, only one of the four included studies employed an ageing model with an age-matched comparison; the remaining three used embryonic neuronal cultures without an ageing comparator. The present review therefore maps the mechanistic basis of NPY-regulated autophagy more comprehensively than it maps how that regulation changes with age. More primary studies on this topic are needed before a robust systematic review could be performed.

### 4.5 Perspectives and future directions

Current evidence supports a neuroprotective role for NPY across multiple age-related neurodegenerative diseases. Nevertheless, significant gaps remain. First, most studies utilise animal models or *in vitro* systems; clinical trials evaluating NPY analogues or gene therapy in humans are lacking. The promising safety of intranasal NPY and the efficacy of vector-mediated delivery warrant early-phase trials to determine optimal dosing, receptor selectivity and long-term outcomes. Second, the short half-life of native NPY limits its therapeutic potential; thus, designing stable NPY analogues, receptor-selective agonists or allosteric modulators with enhanced brain penetration is essential. Third, while NPY stimulates autophagy and mitigates oxidative stress, the downstream signalling pathways linking NPY receptors to autophagy machinery remain poorly understood. Investigating how NPY intersects with mTOR, AMPK and sirtuin pathways could reveal combinatorial strategies to boost autophagy in ageing brains.

## 5 Conclusion

NPY is a neuropeptide closely involved in the mechanisms of brain ageing. Evidence from an accelerated-ageing (progeroid) mouse model shows that hypothalamic NPY declines with ageing and that its restoration produces measurable benefit. It intersects with key pathogenic pathways of brain ageing, oxidative stress, excitotoxicity, inflammation, impaired autophagy and HPA axis dysregulation. While most current evidence supports NPY’s neuroprotective role in mediating ageing-related cognitive decline, further research is needed to fully elucidate its mechanisms and therapeutic potential in humans. Future research should focus on designing studies to thoroughly examine the role of NPY in human brain ageing to validate the findings from animal models. In addition, more research is warranted to investigate the sex-specific effect of NPY in synapse plasticity and also the differential roles of NPY receptors in NPY-mediated neuroprotective mechanisms. Expanding our understanding of NPY’s mechanisms, optimising delivery strategies, and rigorously testing its efficacy and safety in humans will determine whether this peptide can be harnessed as a viable therapy to slow brain ageing and mitigate age-related neurodegenerative disorders.

## Data Availability

The original contributions presented in the study are included in the article/Supplementary material, further inquiries can be directed to the corresponding author.
