# Supplementary material for: Neuropeptide Y and brain ageing: an updated scoping review on the role of autophagy
Source: Front Aging Neurosci. 2026 Sep 21;18:1888512. doi: 10.3389/fnagi.2026.1888512 (PMC13635142; doi:10.3389/fnagi.2026.1888512)
Supplement: Supplementary file 1 [file Table_1.DOCX]

**Supplementary Table S1. Full reproducible electronic search strategies**

Searches were conducted in Web of Science, Scopus, and PubMed for records published from January 2014 to July 2026. The final search was executed on 31 July 2026.

| **Database and platform** | **Full electronic search strategy** | **Search date and limits** | **Records retrieved, n** |
| --- | --- | --- | --- |
| **Web of Science**  *Clarivate Web of Science* | TS=(("Neuropeptide Y" OR NPY) AND ("brain aging" OR "brain ageing"  OR aging OR ageing OR senescence OR "healthy aging" OR "neuronal aging"  OR "cognitive aging") AND (autophagy OR autophagic OR autophagosome*  OR lysosome* OR lysosomal OR mitophagy OR macroautophagy OR microautophagy OR "chaperone-mediated autophagy")) | Date searched: 31 July 2026.  Limits: publication years 2014-2026 and English language. The search date limited the 2026 coverage to records available up to 31 July 2026. No geographical restriction was applied. | **29** |
| **Scopus**  *Elsevier Scopus* | ("Neuropeptide Y"[Mesh] OR "Neuropeptide Y" OR NPY)  AND  ("Aging"[Mesh] OR aging OR ageing OR "Brain Aging" OR "Brain Ageing"  OR senescence OR "healthy aging" OR "brain aging" OR "aging brain"  OR "neuronal aging" OR "cognitive aging")  AND  ("Autophagy"[Mesh] OR autophagy  OR autophagic OR autophagosome* OR lysosome* OR lysosomal OR mitophagy OR macroautophagy OR microautophagy OR "chaperone mediated autophagy") | Date searched: 31 July 2026.  Limits: publication years 2014-2026 and English language. The search date limited the 2026 coverage to records available up to 31 July 2026. No geographical restriction was applied. | **23** |
| **PubMed**  *NCBI PubMed* | TITLE-ABS-KEY  (("Neuropeptide Y" OR NPY)  AND  ("brain aging" OR "brain ageing" OR aging OR ageing OR senescence  OR "healthy aging" OR "healthy ageing"  OR "neuronal aging" OR "cognitive aging")  AND  (autophagy OR autophagic OR autophagosome*  OR lysosome* OR lysosomal  OR mitophagy  OR macroautophagy  OR microautophagy  OR "chaperone-mediated autophagy")  ) | Date searched: 31 July 2026.  Limits: publication date from 1 January 2014 to 31 July 2026 and English language. No geographical restriction was applied. | **17** |
| **Total records retrieved before duplicate removal** | | | **69** |
