# Supplementary material for: Neuropeptide Y and brain ageing: an updated scoping review on the role of autophagy
Source: Front Aging Neurosci. 2026 Sep 21;18:1888512. doi: 10.3389/fnagi.2026.1888512 (PMC13635142; doi:10.3389/fnagi.2026.1888512)
Supplement: Supplementary file 2 [file Table_2.DOCX]

**Supplementary Table S2. Completed PRISMA-ScR Checklist**

*Manuscript: “Neuropeptide Y (NPY) and Brain Ageing: An Updated Scoping Review on the Role of Autophagy”*.

| **Section** | **Checklist item** | **Item** | **PRISMA-ScR reporting requirement** | **Reported on page(s) / location** |
| --- | --- | --- | --- | --- |
| **TITLE** | Title | 1 | Identify the report as a scoping review. | p. 1 (manuscript title) |
| **ABSTRACT** | Structured summary | 2 | Provide a structured summary covering, as applicable, the background, objectives, eligibility criteria, sources of evidence, charting methods, results and conclusions linked to the review questions. | p. 2 (Abstract) |
| **INTRODUCTION** | Rationale | 3 | Explain the rationale in relation to existing knowledge and why a scoping review approach is appropriate. | pp. 3–6 (Introduction; Section 2 opening paragraph) |
| **INTRODUCTION** | Objectives | 4 | State the review questions and objectives explicitly, referring to key elements such as population, concept and context. | p. 6 (Sections 2.1, paragraphs 1–2) |
| **METHODS** | Protocol and registration | 5 | State whether a protocol exists, where it can be accessed and any registration details. | p. 6 (Section 2.2: no protocol registered prospectively) |
| **METHODS** | Eligibility criteria | 6 | Specify eligibility characteristics such as years, language and publication status, and provide a rationale. | p. 7 (Sections 2.3.1–2.3.2) |
| **METHODS** | Information sources | 7 | Describe all information sources, their coverage and the date of the most recent search. | p. 6 (Section 2.2) |
| **METHODS** | Search | 8 | Provide the full reproducible electronic search strategy for at least one database, including limits. | p. 6 (Section 2.2) and Supplementary Table S1 |
| **METHODS** | Selection of sources of evidence | 9 | Describe the screening and eligibility process used to select sources of evidence. | p. 7 (Section 2.3) |
| **METHODS** | Data charting process | 10 | Describe how data were charted, whether forms were calibrated or piloted, whether charting was independent or duplicated and how information was confirmed. | p. 8 (Section 2.4) |
| **METHODS** | Data items | 11 | List and define all variables sought, including assumptions and simplifications. | p. 8 (Section 2.4) and pp. 11–16 (Table 1) |
| **METHODS** | Critical appraisal of individual sources of evidence | 12 | If critical appraisal was performed, explain the rationale, methods and use in synthesis. | p. 8 (Section 2.4: no formal critical appraisal undertaken) |
| **METHODS** | Synthesis of results | 13 | Describe how the charted data were handled, grouped and summarised. | p. 8 (Section 2.4) |
| **RESULTS** | Selection of sources of evidence | 14 | Report numbers screened, assessed and included, reasons for exclusions at each stage and preferably a flow diagram. | pp. 8–9 (Section 3.1; Figure 2) |
| **RESULTS** | Characteristics of sources of evidence | 15 | Present charted characteristics and citations for each included source. | pp. 10–16 (Sections 3.2–3.3; Table 1) |
| **RESULTS** | Critical appraisal within sources of evidence | 16 | If performed, present critical-appraisal findings. | Not applicable; appraisal was not undertaken (see p. 8) |
| **RESULTS** | Results of individual sources of evidence | 17 | Present relevant charted data for each included source in relation to the review questions. | pp. 10–16 (Section 3.3; Table 1) |
| **RESULTS** | Synthesis of results | 18 | Summarise or present charting results in relation to the review questions and objectives. | pp. 10–16 (Sections 3.2–3.3; Table 1) |
| **DISCUSSION** | Summary of evidence | 19 | Summarise the main results, evidence types, concepts and themes; link them to the objectives and consider relevance to key groups. | pp. 17–25 (Sections 4.0–4.3) |
| **DISCUSSION** | Limitations | 20 | Discuss limitations of the scoping-review process. | p. 25 (Section 4.4) |
| **DISCUSSION** | Conclusions | 21 | Interpret the results in relation to the questions and objectives, including implications and next steps. | pp. 25–26 (Section 4.5 and Conclusion) |
| **FUNDING** | Funding | 22 | Report funding for included sources and for the scoping review, together with the role of the review funders. | p. 26 (Funding statement) |

*Checklist source: Tricco AC et al. PRISMA Extension for Scoping Reviews (PRISMA-ScR): Checklist and Explanation. Ann Intern Med. 2018;169:467–473. doi:10.7326/M18-0850.*
